# Novel Derivatives of diphenyl-1,3,4-oxadiazol as Ligands of Benzodiazepine Receptors; Synthesize, Binding Assay and Pharmacological Evaluation

**DOI:** 10.22037/ijpr.2021.115549.15429

**Published:** 2021

**Authors:** Mona Khoramjouy, Naeime Zarepishe, Elham Rezaee, Ali Imani, Rojin Mahmoudzadeh-mandolakani, Seyedali Hashemi, Moones Fallah, Golnar Hasheminasab, Soraya Shahhosseini, Sayyed Abbas Tabatabai, Mehrdad Faizi

**Affiliations:** a *Department of Pharmacology and Toxicology, School of Pharmacy, Shahid Beheshti University of Medical Sciences, Tehran, Iran. *; b *Phytochemistry Research Center, Shahid Beheshti University of Medical Sciences, Tehran, Iran.*; c *Department of Pharmaceutical Chemistry and Radiopharmacy, School of Pharmacy, Shahid Beheshti University of Medical Sciences, Tehran, Iran. *; d *School of Pharmacy, Shahid Beheshti University of Medical Sciences, Tehran, Iran.*; e *Protein Technology Research Center, Shahid Beheshti University of Medical Sciences, Tehran, Iran.*; 1 *M. KH. and N. Z. contributed equally to this work.*

**Keywords:** [3H]-flumazenil, BZD, Radioligand binding assay, Memory, In-vitro, In-vivo

## Abstract

Benzodiazepines (BZD) are among the main classes of tranquilizing drugs, bearing much less toxicity compared to other drugs acting on the CNS. Considering the pharmacophore model of BZD binding to GABA-A receptor, novel diphenyl 1,3,4-oxadiazole compounds as BZD ligands were designed. The compounds were synthesized and structurally confirmed using LCMS, IR and NMR techniques. We investigated the affinity of the compounds to BZD receptors using radioligand [^3^H]-flumazenil by *in-vitro* studies. In addition, sedative-hypnotic, anxiety, anticonvulsant, muscle relaxant, memory impairment, and motor coordination activities of the synthesized compounds were evaluated using *in*-*vivo* studies. Based on *in-vitro* studies, compounds 7i and 7j were the most potent with IC_50_ values of 1.54 and 1.66 nM respectively. *In-vivo* studies showed that compound 7i has the highest impact on increased sedation, muscle relaxation, and decreased anxiety and these observations were antagonized by flumazenil. Compounds 7e and 7i were the most potent anticonvulsant agents among synthesized compounds in both MES and PTZ induced seizure tests. All synthesized compounds significantly decreased latency to fall in the Rotarod test but none of them had a significant impact on the memory impairment test.

## Introduction

Benzodiazepines (BZDs) have been prescribed widely for controlling psychological problems in modern medicine and they have been introduced to medical practice since 1960. Mohler, Okada, Squires, and Braestrup have introduced the benzodiazepine cerebral binding site to GABA-A receptors in 1977 (1, 2). Various types of GABA-A receptors assemble a combination of six different subunits and their isoforms (α1-6; β1-3; ϒ1-3; δ1; ρ1-2 and ε). The most frequent isoform of this pentamer GABA-A receptor consists of α1 (2), β2 (2), and γ2 (1) subunits (3–5). The BZDs act by potentiating GABA-induced chloride currents. The presence of α and ϒl or ϒ2 subunits is an absolute requirement for BZDs binding and optimal modulation of chloride currents by them (6, 7). Although BZDs are the safest psychoactive drugs available today and are widely prescribed as anxiolytics, their sedative and muscle relaxant actions are often considered unwanted side effects. Other unwanted effects of these drugs are amnesia and ataxia. Most BZDs are also associated with anterograde amnesia (8, 9). The pharmacological effects of benzodiazepines (BZDs) make them the most important GABA-A receptor modulating drugs, which are currently used in the clinic (10). Nowadays, agonists of benzodiazepine (BZD) receptor are extensively used in the treatment of epilepsy, anxiety, skeletal muscle spasms and insomnia. Since BZDs have several unwanted effects, including amnesia, cognitive impairment, and ataxia, introducing agonists of the novel BZD receptors can provide potential benefits (11, 12). New BZD receptor ligands with more selective effects such as anti-anxiety and anti-seizure with fewer adverse drug reactions were synthesized in the last two decades. To evaluate the affinity of novel ligands to the binding site of GABA-A receptors, radioligand receptor binding methods are widely being used by investigators. These methods are *in-vitro* techniques and can be used to screen the affinity of ligands to the receptors (13). Medicinal chemistry researchers select the most potent ligands from receptor binding assays for efficacy testing experiments and could further analyze the structure-activity relationship (SAR) for new derivatives at GABA-A BZD receptors. By these kinds of studies, they can discover lead compound(s). In 2005, Selleri *et al.* introduced a group of 2-phenyl-5,7-dimethylpyrazolo[1,5-a] pyrimidine-3-ylacetamides derivatives as ligands of benzodiazepine receptor (14). Furthermore, Tabatabai *et al*. presented novel 2-phenoxy phenyl-1,3,4-oxadiazole compounds as agonists of BZD receptors as hypnotic agents which cause negligible memory weakness (15). Previous evaluations of the structure-activity relationship of ligands of BZD receptors indicated that the ligands should have essential pharmacophores. These pharmacophores are an aromatic ring which is connected to a proton accepting functional group. The group should be coplanar and needs to be located at an appropriate distance (π1) from the aromatic ring. The second essential pharmacophores is another aromatic ring which is out-of-plane of the first aromatic ring and potentiates the binding to the BZD receptor (π3) (16). In this study, diphenyl-1,3,4-oxadiazole derivatives as novel BZD agonists, were designed, synthesized and evaluated based on the pharmacophore/receptor model of BZD binding site of GABA-A receptor (17–20). The *in-vitro* affinity of the synthesized compounds for the central BZD receptor will be determined using radioligand receptor binding assay and subsequently their compounds will be selected for *in-vivo* evaluation. At the end of the study, we hope to develop new compounds as BZD receptor agonists and extend the SAR for ligands. Our aim is to find novel compounds with more selective effect on BZD binding site of GABA-A receptor. These compounds may have a better profile of adverse drug reactions and high efficacy.

## Experimental

*Chemistry*

All laboratory-grade chemicals and solvents were purchased from Merck and Aldrich Companies. Reactions were monitored by thin-layer chromatography (TLC) performed on commercially available Merck precoated plates (silica gel 60 F254, 0.25 mm). Melting points were determined with an electrothermal 9100 unit. Infrared spectra were obtained by a Perkin Elmer 843 IR spectrometer. A Bruker FT-400, 300 MHz (Brucker Biosciences, USA), and Nmready 60 pro, 60 MHz (Nanalysis) devices were used to obtain 1H-NMR spectra with CDCl_3_, DMSO-d6 as solvents and tetramethylsilane (TMS) as internal standard. Coupling constant (J) values are estimated in hertz (Hz) and spin multiples are given as s (singlet), d (doublet), t (triplet), q (quartet), m (multiplet), and br (broad). The mass spectral measurements were performed on an HPLC Agilent system with an electrospray ionization (ESI) interface. 

*Synthesis of ethyl 2-nitrobenzoate (* ***2*** *)*

To a solution of 2-nitrobenzoic acid (5 g, 29.94 mmol) in absolute ethanol (60 mL), concentrated sulfuric acid (6 mL) was slowly added and the mixture was heated under reflux for 24 h. After completion of the reaction, the solvent was evaporated under reduced pressure. The residue was neutralized using NaOH 20% solution and extracted with diethyl ether. Oily liquid, Yield: 76%, IR (KBr):1353 (NO), 1531 (NO), 1727 (C = O), LCMS (ESI): m/z 196 [M+H]^+^.

*Synthesis of 2-nitrobenzohydrazide (* ***3*** *)*

A mixture of ester **2** (4 g, 20.51 mmol) and hydrazine hydrate 98% (1.3 mL, 41.02 mmol) in absolute ethanol was stirred at room temperature. After evaporation of the solvent, the resulting residue was washed with ethanol to give compound **3**. Yellow powder, Yield: 85%, mp: 120-122 C; IR (KBr): 1356 (NO), 1536 (NO), 1635 (C = O), 3177 (NH), 3279 (NH), LCMS (ESI): m/z 204 [M+Na]^+^.

*Synthesis of N’-benzoyl-2-nitrobenzohydrazide (* ***4*** *)*

Compound **3** (3 g, 10.52 mmol) and benzoyl chloride (2.44 mL, 21.05 mmol), in the presence of anhydride sodium carbonate (3.51 g, 33.14 mmol) were reacted in dry dioxane (30 mL) for 24 h at room temperature. After solvent evaporation, the reaction mixture was washed with NaOH 5%, HCl 2M, and water then dried under vacuum to afford compound **4**. White powder, Yield: 95%, m.p: 214-216 °C, IR (KBr): 1352 (NO), 1541 (NO), 1654 (C = O), 1700 (C = O), 3231 (N-H), 3267 (N-H), LCMS (ESI): m/z 308 [M+Na]^+^.

*Synthesis of 2-(2-nitrophenyl)-5-phenyl-1,3,4-oxadiazole(* ***5*** *)*

The mixture of **4** (2 g, 7.02 mmol), thionyl chloride (20 mL) and pyridine was irradiated under the microwave (700 W, 5 min). Ice-cold water was added to the reaction mixture and the formed precipitate was washed and filtered under vacuum to obtain compound **5**. White powder, Yield: 84%, m.p: 131-133 °C, IR (KBr): 1348 (NO), 1521 (NO), LCMS (ESI): m/z 268 [M+H]^+^. 

*Synthesis of 2-(2-Aminophenyl)-5-phenyl-1,3,4-oxadiazole (* ***6*** *)*

SnCl_2_ (6.4 g 33.71 mmol) was added to the solution of the nitro compound **5** (1.5 g, 5.61 mmol) in DMF (10 mL) and stirred for 18 hours at room temperature. The white heavy precipitate was formed upon the addition of water to the reaction mixture. The precipitate was washed with water and filtered under vacuum to afford desired amino compound. Yellow powder, Yield: 81%, m.p: 190-192 °C, IR (KBr): 3238 (NH), 3363 (NH), LCMS (ESI): m/z 260 [M+Na]^+^.

*General procedure for the synthesis of 2,5-diphenyl-1,3,4-oxadiazole derivatives (* ***7a-j*** *)*

A suspension of the amine **6** (0.85 mmol), corresponding acyl chlorides (160 mmol) and anhydride Na_2_CO_3_ (160 mmol) in dry dioxane was stirred for 24 hours at room temperature. Phthalic and succinic anhydrides as acylating agents were used for the formation of compounds **7i** and **7j** respectively and the reaction mixture refluxed in dry toluene. After completion of the reaction, the solid residue was washed with NaOH 20%, HCl 2M, and ice water, and the final compounds were crystallized from ethanol 96%. 

*N-(2-(5-phenyl-1,3,4-oxadiazol-2-yl)phenyl)benzamide (* ***7a)***

Yellow powder, Yield: 81%, m.p.: 188-189 °C, IR (KBr): 1670 (C = O); 3261 (NH) cm^-1^, LCMS (ESI): m/z 340 [M-H]^-^, ^1^HNMR (300 MHz, Chloroform-*d*): 11.86 (broad s, 1H, NH), 9.07 (d, *J* = 8Hz, 1H, H_3_-phenylene), 8.20-7.89 (m, 5H, H_2_, H_6_-benzamido, H_2_, H_6_-phenyl, H_6_-phenylene), 7.59-7.28 (m, 8H, H_3_, H_4_, H_5_-phenyl, H_3_, H_4_, H_5_-benzamido, H_4_, H_5_-phenylene).

*4-chloro-N-(2-(5-phenyl-1,3,4-oxadiazol-2-yl)phenyl)benzamide (* ***7b*** *)*

Yellow powder, Yield: 74%, m.p.: 195-197 °C, IR (KBr): 1689 (C = O); 3323 (NH) cm^-1^, LCMS (ESI): m/z 374 [M-H]^-^, ^1^HNMR (60.16 MHz, Chloroform-*d*): 11.25 (broad s, 1H, NH), 8.2-7.2 (m, 13 H, aromatic).

*4-fluoro-N-(2-(5-phenyl-1,3,4-oxadiazol-2-yl)phenyl)benzamide (* ***7c*** *)*

White powder, Yield: 90%, m.p.: 189-190 °C, IR (KBr): 1674 (C = O); 3312 (NH) cm^-1^, LCMS (ESI): m/z 382 [M+Na]^+^, ^1^HNMR (60.16 MHz, Chloroform-*d*): 11.7 (broad s, 1H, NH), 8.4-7.2 (m, 13 H, aromatic).

*4-methyl-N-(2-(5-phenyl-1,3,4-oxadiazol-2-yl)phenyl)benzamide (* ***7d*** *)*

Yellow powder, Yield: 80%, m.p.: 200-202 °C, IR (KBr): 1671 (C = O); 3305 (NH) cm^-1^, LCMS (ESI): m/z 356 [M+H]^+^, ^1^HNMR (60.16 MHz, Chloroform-*d*): ): 11.5 (s, 1H, NH), 8.1-7.3 (m, 13 H, aromatic) 2.30 (s, 3H, CH_3_). 

*4-nitro-N-(2-(5-phenyl-1,3,4-oxadiazol-2-yl)phenyl)benzamide (* ***7e*** *)*

White powder, Yield: 87%, m.p.: 195-197 °C, IR (KBr): 1348 (NO), 1539 (NO), 1680 (C = O), 3324 (NH) cm^-1^, LCMS (ESI): m/z 387 [M+H]^+^, ^1^HNMR (60.16 MHz, Chloroform-*d*): 11.7 (s, 1H, NH), 8.5-7.5 (m, 13 H, aromatic).

*4-methoxy-N-(2-(5-phenyl-1,3,4-oxadiazol-2-yl)phenyl)benzamide (* ***7f*** *)*

Yellow powder, Yield: 73%, m.p.: 216-217 °C, IR (KBr): 1253 (C-O), 1673 (C = O), 3288 (NH), LCMS (ESI): m/z 372 [M+H]^+^, ^1^HNMR (60.16 MHz, Chloroform-*d*): ): 11.6 (s, 1H, NH), 8.1-7.2 (m, 13 H, aromatic) 3.5 (s, 3H, OCH_3_). 

*N-(2-(5-phenyl-1,3,4-oxadiazol-2-yl)phenyl)acetamide (* ***7g*** *)*

Creamy powder, Yield: 56%, m.p.: 155-157 °C, IR (KBr): 1694 (C = O); 3268 (NH), LCMS (ESI): m/z 280 [M+H]^+^, ^1^HNMR (60.16 MHz, Chloroform-*d*): 9.4 (s, 1H, NH), 8.1-7.3 (m, 9H, aromatic), 2.2 (s, 3H, CH_3_).

*N-(2-(5-phenyl-1,3,4-oxadiazol-2-yl)phenyl) propionamide (* ***7h*** *)*

yellow powder, Yield: 60%, m.p.: 163-165 °C, IR (KBr): 1690 (C = O); 3261(NH), LCMS (ESI): m/z 294 [M+H]^+^, ^1^HNMR (60.16 MHz, Chloroform-*d*): ): 9.6 (s, 1H, NH), 8-7.2 (m, 9H, aromatic), 2.48 (q, *J = *8Hz, 2H, CH_2_), 1.28 (t,* J = *8Hz, 3H, CH_3_),

*2-((2-(5-phenyl-1,3,4-oxadiazol-2-yl)phenyl)carbamoyl)benzoic acid (* ***7i)***

White powder, Yield: 48%, m.p.: 196-198 °C, IR (KBr): 1721 (C = O), 2600-3000 (OH), 3303 (NH) cm^-1^, LCMS (ESI): m/z 384 [M-H]^-^, ^1^HNMR (400 MHz, DMSO-*d*_6_): 13.68 (broad s, 1H, COOH), 10.98 (s, 1H, NH), 8.51 (d, *J* = 8Hz, 1H, H_6_-benzoic acid), 8.12 (d, *J = *8 Hz, 2H, H_2_,H_6-_phenyl), 7.91 (d, *J* = 8Hz, 1H, H_3_-benzoic acid ), 7.75- 7.6 (m, 8H, H_3_, H_5_-phenyl, H_3_, H_4_, H_5_, H_6_-phenylene, H_4_, H_5_-benzoic acid ), 7.41 (t, *J* = 8Hz, 1H, H_4_-phenyl).

*4-oxo-4-((2-(5-phenyl-1,3,4-oxadiazol-2-yl)phenyl)amino)butanoic acid (* ***7j)***

yellow powder, Yield: 64%, m.p.: 159-160 °C, IR (KBr): 1717 (C = O); 2500-3060 (O-H); 3298 (NH), LCMS (ESI): m/z 336 [M-H]^-^, ^1^HNMR (400 MHz, DMSO-*d*_6_): 11.96 (broad s, 1H, COOH), 10.67 (s, 1H, NH), 8.38 (d, *J* = 8Hz, 1H, H_3_-phenylene), 8.16-8.11 (m, 3H, H_2_, H_4, _H_6_-phenyl), 7.69- 7.59 (m, 4H, H_3_, H_5_-phenyl, H_5, _H_6_-phenylene), 7.32 (t, *J* = 8Hz, 1H, H_4_-phenylene), 2.70 (t, *J* = 8Hz, 2H, CH_2_), 2.57 (t, *J* = 8Hz, 2H, CH_2_), ^13^CNMR (400 MHz, DMSO-*d*_6_): 174.58, 171.03, 163.88, 163.74, 137.84, 133.13, 132.73, 129.94, 129.11, 127.27, 127.27,123.52, 121.82, 112.48, 32.32, 29.74. 

*In-vitro studies*

The affinity of the novel synthesized compound to BZD receptors was evaluated by radioligand receptor binding studies utilizing radioligand [^3^H]-Flumazenil through the previously reported procedure by Ahmadi *et al.* (2013). Temporarily, the brain tissue of Male Sprague-Dawley rats (200-250 g weight) were used as the source of the benzodiazepine receptors. These tissues were collected, homogenized in 20 mL of ice‐cold Tris‐HCl buffer and centrifuged at 600 g for 10 min. in the next step, the supernatant was centrifuged 37000xg for 15 min at 4 °C. After that, the final pellet was incubated for 30 min at 37 °C and resuspended in 30 mL Tris‐HCl buffer (50 mM, pH 7.4). The Bradford method using bovine serum albumin as a standard, was applied for quantification of protein in the separated membrane. Saturation and competition experiments are two basic methods of receptor binding studies. In saturation experiment, 100 μg of membrane and various concentrations of [^3^H]-Flumazenil were incubated at 30 °C for 35 min. Saturation experiments were used to measure the receptor binding affinity (Kd) of [^3^H]-Flumazenil and the density of BZD receptors (Bmax). In competition part of the study, 100 μg of membrane protein in Tris.HCl buffer (50 mM, pH 7.4) was incubated with 8.6 × 10-5 nmol [^3^H]-flumazenil and increasing concentrations of the novel synthesized ligands (5 mM–50 pM) for 35 min at 30 °C. Subsequently, the evaluation was completed by centrifugation 1500 g at 4 °C for 5 min. Total bound and nonspecific bound were estimated at various concentrations of non-radioactive ligand. NSB was determined in parallel assays performed in the presence of 100 μM diazepam. Competition experiments were used to measure the percentage inhibition of radioligand specific binding (IC_50_) and affinity (Ki) of the novel synthesized ligands and diazepam, using the Cheng-Prussof equation. These values were determined in comparison with [^3^H]-Flumazenil as a well-known antagonist of BZD receptors (21–23).

*In-vivo studies*

All of the novel synthesized compounds were investigated for their sedative-hypnotic, anti-anxiety, anticonvulsant, muscle relaxant, memory impairment, and motor coordination activities using behavioral responses of mice. In these studies, adult male NMRI albino mice (18–23 g, 6-8 weeks old) were used and obtained from Pasteur Institute, Iran. They were held in eight-mouse cages and housed under standardized conditions in a controlled temperature (22 ± 2 ºC), humidity (50 ± 5 %), light/dark cycle (12 h), and free access to standard diet and water. All the experiments were conducted cautiously based on a protocol approved by the Ethical Committee of Shahid Beheshti University of Medical Sciences and the National Institute for Medical Research development (NIMAD) with approval code IR.NIMAD.REC.1397.481. The animals were randomly divided into different experimental groups.

*Open field test*

To the assessment of the sedative effects and locomotor activities of the novel synthesized compounds, the open field test was performed. Thirty minutes after intraperitoneally administration of the various doses of novel compounds and Diazepam (2 mg/kg), each animal was individually placed at the center of the open field apparatus (40 × 40 × 40 cm). The total distance movement was recorded for 10 min by a digital camera and then was calculated by an automated tracking system (Ethovision XT software, Noldus, The Netherlands) (24).

*Pentobarbital induced sleep test*

To investigation the hypnotic effects of novel synthesized compounds, the pentobarbital induced sleep test was utilized. Thirty minutes after intraperitoneally administration of the different doses of novel compounds and Diazepam (2 mg/kg), Pentobarbital sodium (40 mg/kg IP) was injected for sleep induction. The time between loss and reversal of righting reflex was recorded as sleep duration (25, 26).

*Elevated plus maze test*

To assay the anti-anxiety effects of novel synthesized compounds, the elevated plus-maze test was used. The apparatus of the elevated plus-maze test is prepared of two open arms (30 × 5 × 0.5 cm) and two close arms (30 × 5 × 35 cm) with an open roof and was set at a height of 50 cm above the floor. After pre-treatment of the different doses of novel compounds and Diazepam (2 mg/kg), each animal was individually placed at the middle square of this apparatus and permitted to freely explore the apparatus for 10 min. the time spent in the open arm was recorded by digital stopwatch and the percentage of time spent in open arms was measured (27, 28) 

*Grip strength test*

To assessment the muscle relaxant effects of novel synthesized compounds by measuring the maximum force applied to the digital force meter, the grip strength test was prepared. This assessment was guided using the modified method explained by Bachstetter *et al.* (2014). The test was repeated 3 times for each mouse, 30 min after injection of the novel compounds (29).

*Maximal electro shock (MES) and Pentylenetetrazol (PTZ) tests*

Two useful seizure models (MES and PTZ) were organized for evaluation of the anticonvulsant activities of novel synthesized compounds. In these experiments, the capability of novel synthesized compounds to prevent the maximal electro shock-induced seizure and pentylenetetrazol-induced seizure were screened. MES test (60 Hz, 50 mA, 0.2 s) was done according to the method described by Toolabi *et al.* (2020). PTZ test was done Using the mothed described by Ranjbar-ekbatan *et al.* (2019). These experiments were applied 30 min after administration of the novel compounds. The number of HLTE (hind limb tonic extension) in the MES test and the number of dead animals in the PTZ test were counted (30–32).

*Rotarod test*

For evaluation of the motor coordination and balance capability, the rotarod test was used. Concisely, 30 min after injection of the novel compounds and Diazepam (2 mg/kg), each animal was located on a rotating rod (6 rpm, 1 min). In this experiment, the average time latency to fall for each animal was noted (33).

*Passive avoidance test*

To determine the effects of novel synthesized compounds on learning and memory disorder, the passive avoidance test was done. The passive avoidance apparatus consists of two compartments, that one of them is brightly lit (white compartment) and another one is dimly lit (black compartment). A sliding door divides these two compartments. During the initial phase, after administration of the different doses of novel compounds and Diazepam (2 mg/kg), each animal was located in the white compartment. After arriving in the black compartment, it was received a mild unpleasant electrical stimulation (0.5 mA for 2 s) through the grid floor. During the test phase, the animal was relocated in the white compartment. However, there was no electric stimulation in the dark compartment. The latency to enter the dark compartment was recounted (34,35).

*Statistical analysis*

In this study, we analyzed all the results by Graph Pad Prism Software (V. 9) and presented them as significant at *P* < 0.05. All values were represented as mean (with 95% confidence intervals). To determine the IC_50_ values *in-vitro* studies non-linear regression methods were used. To determine the ED_50_ values *in-vivo* studies, non-linear regression and chi-square and fisher’s exact methods were used. All data were analyzed by one-way analysis of variance (one-way ANOVA) and Tukey’s post hoc tests. 

## Results and Discussion

*Chemistry*

**Scheme 1:**
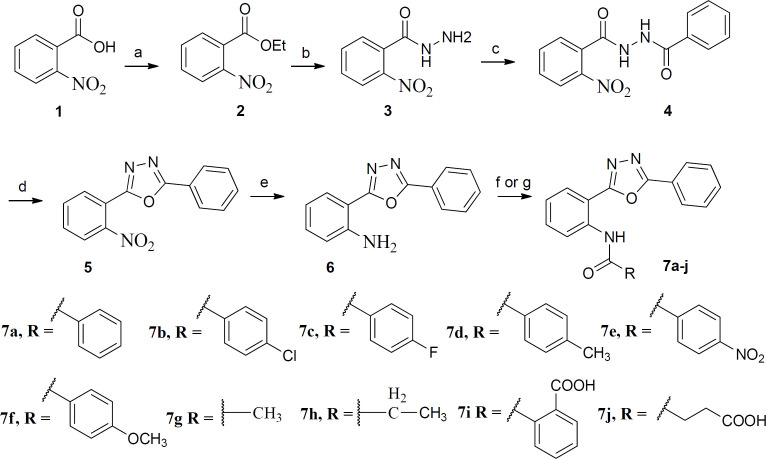
Reagents and conditions: (a): conc. H_2_SO_4_, absolute ethanol, reflux, 24 h; (b): NH_2_NH_2_, absolute ethanol, rt, 18 h; (c): Benzoyl chloride, anhydrous Na_2_CO_3_, dry dioxane, rt, 14 h; (d): SOCl_2_, pyridine, microwave, 5 min; (e): SnCl_2_, DMF, rt, 18 h; (f): Benzoyl chlorides, anhydrous Na_2_CO_3_, dry dioxane, rt, 18- 48 h; (g): Phthalic anhydride or Succinic anhydride, toluene, reflux, 72 h

Diphenyl-1,3,4-oxadiazole derivatives **7a-j** were synthesized according to Scheme 1. Esterification of 2-nitrobenzoic acid with ethanol afforded compound **2** which was treated with hydrazine hydrate to obtain compound **3.** Oxadiazole **5 **was prepared by the reaction of compound **3** with benzoyl chloride followed by treatment with SOCl_2_ and pyridine under microwave conditions. The nitro group reduced by SnCl_2 _and final compounds **7a-j **were synthesized by reaction of the amine **6** with benzoyl chlorides and carboxylic acid anhydrides in dioxane and toluene respectively. 

*In-vitro* *studies*

In saturation studies, the K_d_ and B_max_ are 1.253 ± 0.344 nM and 5.263х10-4 ± 4.689х10-5 nmol/mg, respectively. Based on the competition studies, the results of IC_50_ (half-maximal inhibitory concentration) and K_i_ (binding affinities of the ligands) are summarized in Table 1. Several novel agents in the table showed appropriate affinity to BZD receptors of rat brain compared to diazepam, as a standard agonist of BZD receptor (Table 1). In this series, compounds ***7i*** (R = 2-Carboxyphenyl) and **7j** (2-carboxyethyl) with electron-withdrawing substituent in R position which have IC_50 _values of 1.54 and 1.66 nM respectively, are the most potent analogs. These values were comparable with the affinity of diazepam to the BZD receptors (IC_50_ = 1.71 nM and Ki = 0.99 nM). The rank order for the contribution of substituents on phenyl ring is: OCH_3_> CH_3_ > F > Cl > NO_2_> H. It seems that the presence of substituent on phenyl ring improved affinity to the benzodiazepine receptor.

*In-vivo studies*

The results of *in-vivo* studies were depicted as ED_50_ values presented in Table 2. In the open field test, all compounds were less potent compared to diazepam (ED_50_ = 1.69 mg/kg. Both Compound **7i** (ED_50_ = 7.14 mg/kg) and **7j** (8.90 mg/kg), bearing carboxyl group, decreased the total distance movement and had the highest sedative effect (Table 2). 

In the pentobarbital-induced sleep test, the hypnotic effects of the novel compounds were weaker than diazepam with an ED_50_ value of 1.98 mg/kg. considering the impact of different substitutions on this test compounds **7i** and **7j** with polar carboxyl group showed the highest sleeping effects with ED_50_ values of 4.64 and 4.98 mg/kg respectively (Table 2).

In the elevated plus-maze test, Diazepam with an ED_50_ value of 2.24 mg/kg increased the percentage of time spent in open arms. Compounds with polar substitutions, **7i** (R = 2-carboxy phenyl), **7j** (2-carboxy ethyl) and **7e** (4-nitro phenyl), were the most potent analogs with ED_50_ values of 8.71 mg/kg, 9.77 mg/kg and 9.96 respectively (Table 2).

In the grip strength test, Diazepam with an ED_50_ value of 2.27 (1.474 to 3.481) mg/kg decreased the recorded forces applied to the grip strength device. According to the results obtained from all mentioned tests, the most favored results were observed with polar compounds **7i **and **7j** with ED_50_ values of 13.15 mg/kg and 17.07 mg/kg. 

The sedative-hypnotic, anti-anxiety, and muscle relaxant effects of the novel synthesized compounds were significantly inhibited by flumazenil (10 mg/kg) as a standard antagonist of BZD receptors (*P* < 0.001). It means that the novel synthesized compounds show their effects through BZD receptors.

Compounds **7e** and **7i** both bearing polar electron-withdrawing groups of 2-carboxyl and 4-nitro showed significant anticonvulsant activities with the ED_50 _values of 24.16 and 26.74 mg/kg in the MES test and 45.25 and 45.12 mg/kg in the PTZ test, respectively. As shown in Table 2, Compound **7a** (R = phenyl), has the least activity in all tests. 

**Figure 1:**
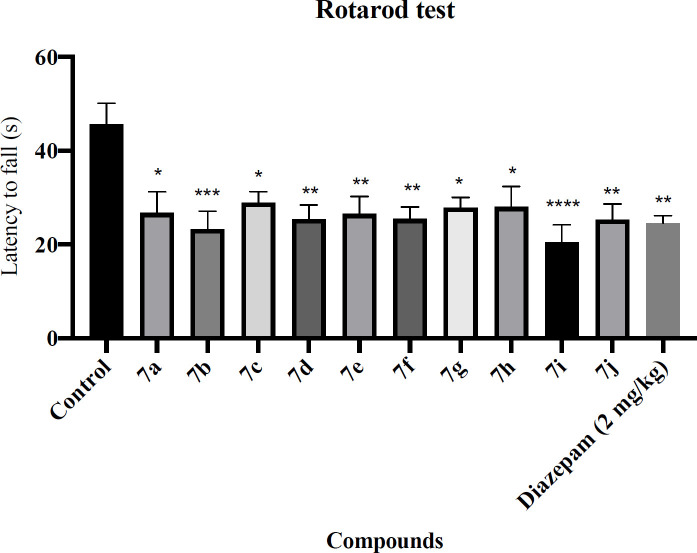
Effect of the novel compounds on motor coordination in rotarod test; The latency time to fall (s) are shown. Diazepam (2 mg/kg) was used as a positive control. The results were analyzed by one-way ANOVA followed by Tukey’s test. Data are presented as mean ± SEM. ^*^represents *P* < 0.5, ^**^represents *P* < 0.01, ^***^represents *P* < 0.001 and ^****^represents *P* < 0.0001 compared to the control group. n = 8 in all groups

**Figure 2:**
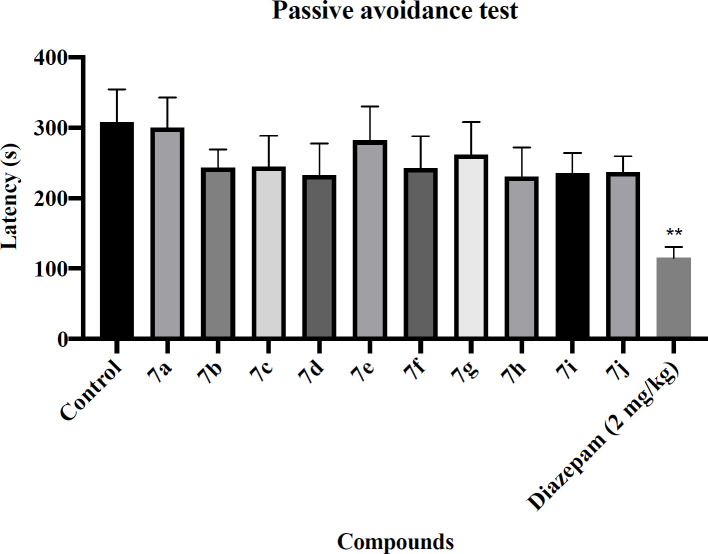
Effect of the novel compounds on memory function in passive avoidance test; The latency time to enter the dark compartment in the testing day (s) are shown. Diazepam (2 mg/kg) was used as a positive control. The results were analyzed by one-way ANOVA followed by Tukey’s test. Data are presented as mean ± SEM. ^**^represents *P* < 0.01 compared to the control group. n = 8 in all groups

The rotarod and passive avoidance tests were used to evaluate the side effects of the novel synthesized compounds. The results of the rotarod test are shown in Figure 1. Diazepam showed a significant decrease in latency to fall compared with the control group (*P* < 0.01). Regretfully, all of the novel synthesized compounds were significantly decreased latency to fall. The results of the passive avoidance test are shown in Figure 2. Diazepam showed a significant reduction in latency to enter the dark compartment compared with the control group (*P* < 0.01), denoting anterograde amnesia. The avoidance latency did not change in any group of novel synthesized compounds compared with the control group. It means that these novel synthesized compounds had no adverse effect on memory function. 

**Figure 3:**
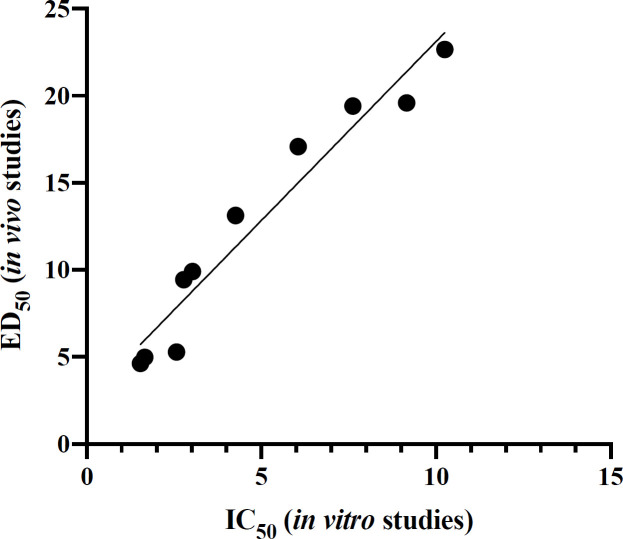
Diagram of the correlation of obtained IC_50_ of *in-vitro* and ED_50_ of *in-vivo* studies of the novel synthesized compounds. This correlation was highly significant (R2 = 0.9401; *P* < 0.0001)

Interestingly, there is a direct correlation between the IC_50_ values in *in-vitro* studies and the ED_50_ values of pentobarbital induced sleep tests in *in-vivo* studies. There is a great correlation (R^2 ^= 0.9401, *P* < 0.0001) between the affinity of the compounds to the BZD receptor and their hypnotic activity (Figure 3).

**Table 1:** *In-vitro* binding affinities of the novel compounds in competition study using [^3^H]-flumazenil binding to benzodiazepine receptor

**Compound**	**IC**_50_ ** (95% CI)** **(nM)**	**K**_i_ **(nM)**
**7a**	10.25 (3.13 to 33.49)	5.95 (1.81 to 19.47)
**7b**	7.62 (4.39 to 13.23)	4.43 (2.55 to 7.69)
**7c**	6.05 (1.93 to 19.01)	3.51 (1.12 to 11.05)
**7d**	4.26 (1.44 to 12.63)	2.47 (0.83 to 7.34)
**7e**	9.16 (4.57 to 18.36)	5.32 (2.65 to 10.67)
**7f**	3.03 (0.65 to 14.0)	1.76 (0.37 to 8.13)
**7g**	2.78 (0.93 to 8.30)	1.61 (0.54 to 4.82)
**7h**	2.57 (0.62 to 10.57)	1.49 (0.36 to 6.14)
**7i**	1.54 (0.65 to 3.64)	0.89 (0.26 to 2.80)
**7j**	1.66 (0.81 to 3.38)	0.96 (0.47 to 1.96)
**Diazepam**	1.71 (0.84 to 3.46)	0.99 (0.48 to 2.01)

**Table 2:** Results of *in vivo *pharmacological tests of the novel synthesized compounds

**ED**_50_ ** (95% CI) (mg/kg)**						
**Grip strength test**	**PTZ test**	**MES test**	**Elevated plus maze test**	**Pentobarbital induced Sleeping test**	**Open field test**	**Compound**
34.89 (28.72 to 42.71)	276.2 (164.2 to 606.9)	234.7 (163.8 to 370.6)	15.34 (12.02 to 19.55)	22.67 (18.07 to 28.45)	27.71 (22.32 to 34.41)	**7a**
31.48 (24.80 to 40.32)	179.8 (102.7 to 396.5)	162.6 (99.41 to 310.0)	11.81 (9.51 to 14.48)	19.41 (12.76 to 29.54)	21.28 (15.08 to 30.04)	**7b**
30.89 (25.36 to 37.87)	118.2 (60.82 to 294.1)	105.1 (60.73 to 209.1)	13.73 (10.08 to 18.23)	17.09 (10.53 to 27.77)	19.71 (17.28 to 22.48)	**7c**
29.39 (24.17 to 35.92)	108.8 (61.03 to 226.8)	92.46 (49.57 to 202.3)	12.56 (9.84 to 15.65)	13.12 (9.94 to 17.31)	18.81 (16.16 to 21.88)	**7d**
25.74 (21.30 to 31.20)	45.25 (26.34 to 80.29)	24.16 (18.27 to 31.87)	9.96 (7.94 to 12.22)	19.60 (13.48 to 28.49)	19.15 (13.52 to 27.14)	**7e**
26.74 (23.15 to 30.95)	135.5 (92.44 to 214.7)	135.5 (92.44 to 214.7)	12.72 (9.94 to 16.01)	9.91 (7.62 to 12.88)	14.50 (9.59 to 21.92)	**7f**
28.84 (24.54 to 34.01)	146.8 (56.17 to 1004)	186.1 (99.76 to 482.6)	12.79 (9.41 to 16.95)	9.45 (9.59 to 21.96)	14.03 (10.07 to 19.56)	**7g**
24.64 (20.18 to 30.17)	135.5 (92.44 to 214.7)	118.2 (60.82 to 294.1)	11.76 (9.59 to 14.22)	5.28 (4.72 to 5.91)	10.05 (4.38 to 23.05)	**7h**
13.15 (11.45 to 15.07)	45.12 (20.82 to 106.0)	26.74 (6.945 to 104.4)	8.71 (7.06 to 10.50)	4.64 (3.44 to 6.25)	7.14 (5.46 to 9.34)	**7i**
17.07 (14.49 to 20.09)	60.86 (31.53 to 129.8)	40.81 (16.91 to 107.8)	9.77 (8.04 to 11.67)	4.98 (4.55 to 5.45)	8.90 (7.52 to 10.55)	**7j**
2.27 **(**1.474 to 3.481)	1.01 (0.21 to 2.49)	1.32 (0.50 to 3.36)	2.24 (1.41 to 2.99)	1.98 (1.75 to 2.25)	1.69 (1.45 to 1.98)	**Diazepam**

## Conclusion

Novel derivatives of diphenyl 1, 3, 4-Oxadiazole as agonists of benzodiazepine receptors were investigated. Some of the novel synthesized compounds showed a better affinity for the BZD site of action on the GABA-A receptor complex than Diazepam in radioligand receptor binding assay. All of the novel synthesized derivatives were evaluated for pharmacological assays. Surprisingly, a desirable correlation was observed between the ED_50_ values in the pharmacological evaluation and IC_50_ values in the radioligand receptor binding assay. Compounds **7i **and** 7j **with polar withdrawing substituent exhibited proper hypnotic, sedative, and muscle relaxant activities. All of the novel synthesized compounds showed no adverse effect on memory function. Memory deficit is an important unwanted effect of some BZDs. All of the novel derivatives had almost no significant negative effect on learning and memory in this study. Therefore, the novel derivatives could be the lead compounds in designing novel BZD ligands in future studies. Previous studies indicated that the α_1_ subunit of the BZD receptor is the most important subunit in the sedative-hypnotic (and partly the anti-seizure) effects of BZD agonists. In addition, we know that the α_5_ subunit is highly involved in the memory defect caused by BZDs. Therefore, we can conclude that compound **7i** has probably more affinity to the α_1_ subunit of GABA-A receptors in comparison with the α_5_ subunit. However, this suggestion requires additional experiments to be confirmed. 

## Declaration of Interest

The authors declare that they have no Conflict of Interest. 
